# Assessment of vitamin D status and parathyroid hormone during a combined intervention for the treatment of childhood obesity

**DOI:** 10.1038/s41387-019-0083-z

**Published:** 2019-06-04

**Authors:** Teodoro Durá-Travé, Fidel Gallinas-Victoriano, María Jesús Chueca-Guindulain, Sara Berrade-Zubiri, María Urretavizcaya-Martinez, Lotfi Ahmed-Mohamed

**Affiliations:** 1 Department of Pediatrics, School of Medicine, University of Navarra, Pamplona, Spain; 2 Department of Pediatrics, Navarra Hospital Complex, Pamplona, Spain; 3 Navarra Institute for Health Research (IdisNA), Pamplona, Spain

**Keywords:** Endocrinology, Endocrine system and metabolic diseases

## Abstract

**Background:**

Obesity is associated with vitamin D deficiency. The aim of this work is to analyze the changes in vitamin D status and PTH levels in a group of children with obesity receiving combined intervention program in order to get BMI status reduction.

**Methods:**

Longitudinal study in 119 children with obesity, aged 9.1–13.9 years, included in a 1-year combined dietary-behavioral-physical activity intervention. Anthropometric measurements (weight, height, BMI and fat mass index) were registered every 3 months and blood testing (calcium, phosphorous, 25(OH)D and PTH) were collected at the beginning and after 12 months of follow-up. A control group was recruited (300 healthy children, aged 8.1–13.9 years). The criteria of the US Endocrine Society were used for the definition of hypovitaminosis D.

**Results:**

Vitamin D deficiency was significantly higher in obesity group (31.1 vs. 14%). There was negative correlation between 25(OH)D and fat mass index (*r* = −0.361, *p* = 0.001). Patients with BMI reduction throughout combined intervention were 52 (43.7%). There was a significant increase in the prevalence of hypovitaminosis D in patients without BMI reduction at the end of follow-up, but in those patients with BMI reduction there was no changes of vitamin D status.

**Conclusions:**

Obesity increases the prevalence of suboptimal vitamin D status, and a BMI status reduction in children with obesity may be required to at least stabilize vitamin D status.

## Introduction

Obesity is associated with vitamin D deficiency^1–4^. The main source of vitamin D for humans is the exposure to natural sunlight and, therefore, the major cause of vitamin D deficiency is inadequate sunlight exposure^5^. Therefore, the higher prevalence of vitamin D deficiency in children with obesity might be related to a more sedentary lifestyle and, consequently, a decreased sun exposure.

However, the reason for the association between obesity and lower levels of 25-hydroxy vitamin D (25(OH)D) and higher parathyroid hormone (PTH) levels is still unclear, and several hypotheses have been proposed about this relationship. The low levels of 25(OH)D in obesity have been attributed to excessive fat-soluble vitamin deposition in adipose tissue and, consequently, a decreased bioavailability of vitamin D^6^. On the other hand, secondary elevation of PTH has been postulated as a predictor of obesity^7^. PTH might increase calcium influx into adipocytes and thus promote lipogenesis and weight gain^8,9^. Additionally, some studies support that vitamin D increases and PTH decreases after weight loss in both obese adults^10^ and children^11^, suggesting that these changes would be more a consequence than the cause of overweight.

Gender, age, season of the year in which blood is collected and place of residence have also been associated with vitamin D deficiency in healthy school-age children and adolescents^2,12,13^. However, it is unclear whether the same factors could be associated with hypovitaminosis D in children and adolescents with obesity.

Present day society has witnessed a progressive increase in the prevalence of childhood obesity over the last decades. The treatment for this condition is complex and has inconclusive results so far but, after the application of combined strategies, it might turn to be effective^14–18^.

The aim of this study is to analyze the changes in vitamin D status and PTH levels in a group of children with obesity included in a combined dietary-behavioral-physical activity intervention program in order to get BMI status reduction.

## Methods

### Participants

The work presented is a longitudinal study carried out in a sample of 119 patients diagnosed with obesity (59 boys and 60 girls), aged 9.1–13.9 years (median: 11.5 years). All patients included in the study were Caucasian and passed a clinical examination (clinical evaluation was performed every 3 months thereafter) and blood testing before and after the participation in a 1-year intervention program that contains a combination of dietary-behavioral-physical activity. The attention was provided in the Pediatric Endocrinology Unit of the Navarra Hospital Complex (Pamplona, Spain), in the period January 2016-December 2017. They did not receive any additional vitamin D or calcium supplements throughout the combined intervention or previously. Pubertal stage was assigned according to Tanner’s criteria in every patient. Children in Tanner stage I were considered prepubertal, and those in Tanner stages II–V were considered pubertal. The place of residence was characterized as urban or rural by means of population (more or less than 10,000 inhabitants, respectively). Overweight individuals were not included in this study.

In addition to that, these parameters (clinical examination and blood testing) were determined in a control group that consisted of 300 healthy Caucasian children (132 boys and 168 girls), aged 8.1–13.9 years (median: 11.2 years), with normal nutritional status: BMI *z*-score ranging from −1.0 (15th percentile) to +1.0 (85th percentile). These participants came from external consultations of the different pediatric subspecialties. There were not any participants suffering from any illness affecting bone health or chronic pathologies that might interfere growth, body composition, food ingestion or physical activity, nor had received any medication (antiepileptic drugs or glucocorticoids) and vitamin D or calcium supplements.

### Clinical examination

The standardized protocol that was used for the anthropometric measurements has been previously published^18^. Anthropometric measurements in patients with obesity were recorded after the first consultation and every 3 months: weight, height, body mass index (BMI) and skinfold thickness (biceps, triceps, sub scapular and suprailiac) and waist circumference.

Weight and height measurements were taken with subjects wearing undergarments and barefoot. Weight was measured using an Año-Sayol scale (reading interval 0–120 kg and a precision of 100 g), and height was measured using a Holtain wall stadiometer (reading interval 60–210 cm, precision 0.1 cm). BMI was subsequently calculated by means of the following formula: weight (kg)/height^2^ (m).

Skinfold thickness (triceps, biceps, sub scapular, and suprailiac) values were measured with an accuracy of 0.1 mm on the left side of the body with Holtain skinfold caliper (CMS Weighing Equipment, Crymych, United Kingdom). The body fat percentage (%) and fat mass (kg) were determined by means of the equations reported by Siri et al., adjusted for sex and age^19^. In the same way, the fat mass index was calculated using the following formula: fat mass (kg)/height^2^ (m). Waist circumference was registered using a tape measure placed on a horizontal line equidistant from the last rib and the iliac crest. Measurements were performed by the same trained individual.

The *z*-score values for the BMI and skinfold thickness and waist circumference were calculated applying the program Aplicación Nutricional, from the Spanish Society of pediatric gastroenterology, hepatology and nutrition (Sociedad Española de Gastroenterología, Hepatología y Nutrición Pediátrica, available at http://www.gastroinf.es/nutritional/). The graphics from Ferrández et al. (Centro Andrea Prader, Zaragoza 2002) were used as reference charts^20^.

Participants were secondarily diagnosed with obesity when *z*-score values for the BMI were higher than 2.0 (97th percentile).

### Blood testing

Fasting calcium and phosphorus plasma levels were measured by colorimetric methods using a COBAS 8000 analyzer (Roche Diagnostic, Mannheim, Germany). 25(OH)D levels were calculated by a high-specific chemiluminiscence-immunassay (LIAISON Assay, Diasorin, Dietzenbach, Germany), and PTH levels were determined by a highly specific solid-phase, two-site chemiluminescent enzyme-labeled immunometric assay using an Immulite analyzer (DPC Biermann, Bad Nauheim, Germany).

The criteria for classification of vitamin D status of the United States Endocrine Society were used for categorization. Vitamin D deficiency was then defined when 25(OH)D levels were lower than 20 ng/ml (<50 nmol/L), Vitamin D insufficiency when 25(OH)D levels were between 20 and 29 ng/ml (50–75 nmol/L) and Vitamin D sufficiency when 25(OH)D levels reached or surpassed 30 ng/ml (>75 nmol/L)^21,22^. Secondary hyperparathyroidism was considered when PTH serum levels were higher than 65 pg/ml^2,7^.

### Combined dietary-behavioral-physical activity intervention

The combined intervention has been previously explained^18^. The central idea of the program is based on the axiom: “the child becomes skinny keeping a stable weight because he/she is growing” and it included nutritional education, a nutritional intervention, the promotion of physical activity and healthy lifestyles and self-monitoring on body weight (weekly registration of weight).

All the patients were required to acquire basic practical and theoretical concepts adequate to enable self-monitoring in order to be included in this study. A multidisciplinary team (nurse, pediatrician and nutritionist) instructed the patients and their families in nutritional education, synchronizing the education and the first visit. The contents of these structured sessions (nutritional value of the different food groups, food pyramid, physical activity, etc.) were customized according to the characteristics of each patient and/or family and continuous guidance was provided to all of them. The program was developed or extended depending on the needs of the patient in subsequent visits.

The approach to maintaining the actual weight is reached by means of a diversified and well-balanced diet for the whole family with no strict restrictions or immediate or exaggerated weight loss. The Mediterranean diet, adapted to family customs or the preferences of the participants, was the model diet. Five daily meals were mandatory, with the condition that meal schedules were respected. The participants were taught to avoid eating in between meals and to increase the time of intake (eating slowly and chewing the food).

In addition, a personalized planning to increase physical activity was suggested to each individual and consisted of a daily, regulated (60 min) free-choice activity (cycling, swimming, martial arts, walking, etc.) and an increase in daily activity (such as walking, avoiding the use of the elevator, helping in house hold chores, etc.).

A leaflet with general recommendations for their daily diet, physical activity (sports and home activity) and healthy lifestyle was handed to every family.

A good response to treatment (BMI status reduction) was reported when a BMI *z*-score reduction greater than or equal to 0.25 units of the initial value occurred after 12 months of follow up; otherwise, it was considered a failure to treatment^23^.

### Statistical analysis

Results are presented as percentages (%) and means (M) with the corresponding standard deviations (SD). A posterior statistical analysis (descriptive statistics, Chi-square test and Pearson’s correlation, Student’s *t*-test or paired *t*-test) was executed using the program Statistical Packages for the Social Sciences version 20.0 (Chicago, IL, USA). Statistical significance was accepted when *P*-value was <0.05.

Parents and/or legal guardians were informed and provided consent for the participation in this study in all cases. This study was approved by the Ethics Committee for Human Investigation of our institution (in accordance with the ethical standards laid down in the 1964 Declaration of Helsinki and later amendments).

## Results

Table 1 shows and compares the distribution of the presumed risk factors for hypovitaminosis D between obesity and control groups. There were no significant differences in the distribution in relation to sex, age group, season of blood sample collection and place of residence.

**Table 1:** Distribution of presumed risk factors for hypovitaminosis D in obesity and control groups

Items	Obesity group (*n* = 119)	Control group (*n* = 300)	*P*-value
Sex			
Boys	59 (49.6%)	132 (44%)	0.562
Girls	60 (50.4%)	168 (56%)	
Age group			
Childhood	35 (29.4%)	99 (33%)	0.636
Adolescent	84 (70.6%)	201 (67%)	
Season of study visit			
Winter	28 (23.5%)	69 (27.6%)	0.541
Spring	24 (20.2%)	57 (22.8%)	
Summer	38 (31.9%)	54 (21.6%)	
Autumn	29 (24.4%)	70 (28.0%)	
Residence			
Urban	57 (47.9%)	181 (60.3%)	0.342
Rural	62 (52.1%)	119 (39.7%)	

Table 2 shows and compares mean values of clinical and biochemical characteristics registered between obesity and control groups before the combined intervention. Weight *z*-score, height *z*-score, BMI *z*-score, body fat percentage, fat mass, fat mass index and waist circumference *z*-score were significantly higher in children with obesity (*P* < 0.05). 25(OH)D levels were significantly higher in the control group (*P* < 0.05), whereas the mean values for PTH levels were significantly higher in the obesity group (*P* < 0.05). There were no significant differences in age, calcium and phosphorus levels among both groups.

**Table 2:** Clinical and biochemical characteristics in obesity and control groups before combined intervention (M ± DS)

Items	Obesity group (*n* = 119)	Control group (*n* = 300)	*P*-value*
Age (y)	11.6 ± 2.3	11.2 ± 2.0	0.171
Weight *z*-score	3.1 ± 1.3	−0.2 ± 0.7	0.001
Height z-score	0.7 ± 1.0	0.04 ± 1.1	0.001
BMI z-score	3.1 ± 1.2	−0.06 ± 0.4	0.001
Percentage body fat (%)	37.6 ± 4.6	24.9 ± 6.0	0.001
Fat mass (kg)	26.6 ± 7.1	9.9 ± 3.0	0.001
FMI (kg/m²)	10.8 ± 1.9	4.7 ± 0.3	0.001
WC *z*-score	2.5 ± 1.2	−0.4 ± 0.7	0.001
Calcium (mg/dL)	9.9 ± 0.3	10.0 ± 0.3	0.891
Phosphorus (mg/dL)	4.5 ± 0.5	4.5 ± 0.5	0.904
25(OH)D (ng/mL)	21.9 ± 8.1	27.9 ± 7.5	0.001
PTH (pg/mL)	51.7 ± 17.0	31.4 ± 13.8	0.001

*Student’s *t*-test

*BMI* body mass index, *FMI* fat mass index, *PTH* parathyroid hormone, *WC* waist circumference

**Fig. 1:**
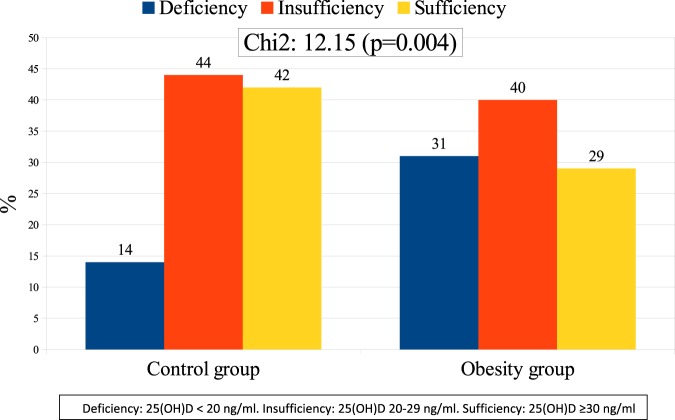
Vitamin D status in control and obesity groups before combined intervention

Figure 1 depicts and compares the prevalence of hypovitaminosis D between obesity and control groups before combined intervention. Hypovitaminosis D prevalence was significantly higher in the obesity group (insufficiency: 40.3% and deficiency: 31.1%) than in the control group (insufficiency: 44% and deficiency: 14%). Likewise, the frequency of hyperparathyroidism was significantly higher in the obesity group (20.2%) than in the control group (4%).

Table 3 compares the prevalence of each vitamin D status in relation to the presumed risk factors for hypovitaminosis D in the obesity group. There were no significant differences in relation to sex and age group, but individuals living in an urban environment showed a significantly higher prevalence of vitamin D deficiency compared to those living in rural environment. In addition, the prevalence of vitamin D deficiency was significantly higher in winter, spring and autumn compared to summer.

**Table 3:** Prevalence of vitamin D status according to the presumed risk factors for hypovitaminosis D in patients with obesity

Item	Deficiency *n* (%)	Insufficiency *n* (%)	Sufficiency *n* (%)	χ^2^, p-value
Sex				
Boys	14 (23.7)	28 (47.5)	17 (28.8)	0.169
Girls	23 (39.0)	21 (35.0)	16 (27.1)	
Age group				
Childhood	9 (25.7)	18 (51.4)	8 (22,9)	0.304
Adolescent	28 (33.3)	31 (36.9)	25 (27.8)	
Residence				
Urban	28 (49.1)	18 (31.6)	11 (19.3)	0.010
Rural	12 (19.3)	29 (46.8)	21 (33.9)	
Season of study visit				
Winter	11 (39.3)	12 (42.9)	5 (17.9)	0.018
Spring	8 (33.3)	10 (41.7)	6 (25.0)	
Summer	7 (18.4)	12 (31.6)	19 (50.0)	
Autumn	11 (37.9)	14 (48.3)	4 (13.8)	

*χ*^2^: Chi square test

At baseline, there was statistically significant negative correlation between 25(OH)D and weight z-score (*r* = −0.241, *P* = 0.012), BMI *z*-score (*r* = −0.272, *P* = 0.005), fat mass (*r* = −0.271, *P* = 0.007), fat mass index (*r* = −0.361, *P* = 0.001), waist circumference *z*-score (*r* = −0.210, *P* = 0.03) and PTH (*r* = −0.320, *P* = 0.012), but no significant correlation to percentage body fat, calcium or phosphorus. There was statistically significant positive correlation between PTH and fat mass (*r* = 0.293, *P* = 0.004), fat mass index (*r* = 0.341, *P* = 0.001), but no significant correlation to weight *z*-score, BMI *z*-score, waist circumference *z*-score, percentage body fat or phosphorus. In addition, there was statistically significant negative correlation between PTH and calcium (*r* = –0.281, *P* = 0.004).

A reduction of BMI status in the obesity group between the beginning and the end of the combined intervention (12 months) was observed in 52 patients (43.7%), and there were no statistically significant differences in relation to sex, age group, season of blood sample and place of residence.

Table 4 shows and compares mean values of anthropometric and biochemical characteristics registered in obesity group with and without reduction of BMI status throughout combined intervention (at the beginning and after 12 months of follow-up). In patients with reduction of BMI status the weight *z*-score, BMI *z*-score, percentage body fat, fat mass index, and waist circumference *z*-score significantly decreased (*P* < 0.05) along the follow-up period. However, in patients without reduction of BMI status, there were no significant differences in weight *z*-score, BMI *z*-score, percentage fat mass, fat mass, fat mass index, and waist circumference *z*-score throughout combined intervention. Throughout combined intervention (at the beginning and after 12 months of follow-up), there were no significant differences in the levels of calcium, phosphorous, 25(OH)D and PTH both in patients with BMI status reduction and in patients without BMI status reduction.

**Table 4:** Changes in anthropometric and biochemical characteristics in obesity group with and without BMI status reduction throughout combined intervention

Items	With BMI status reduction (*n* = 52)			Without BMI status reduction (*n* = 67)		
	At baseline	1 year later	*P*-value*	At baseline	1 year later	*P*-value*
Weight (kg)	69.9 ± 13.4	68.4 ± 13.4	0.529	69.7 ± 14.1	77.3 ± 16.1	0.035
Weight *z*-score	3.3 ± 1.4	2.5 ± 1.2	0.005	3.2 ± 1.3	3.4 ± 1.3	0.588
Height (cm)	153.4 ± 11.4	158.6 ± 9.9	0.019	154.3 ± 11.4	159.8 ± 12.3	0.062
Height *z*-score	0.7 ± 1.1	0.7 ± 1.1	0.206	0.9 ± 1.1	0.8 ± 1.1	0.592
BMI *z*-score	3.4 ± 1.3	2.5 ± 1.1	0.002	3.2 ± 0.9	3.3 ± 1.1	0.474
Percentage body fat (%)	38.6 ± 4.6	35.0 ± 4.1	0.006	38.8 ± 4.7	39.0 ± 3.9	0.184
Fat mass (kg)	27.0 ± 7.1	25.2 ± 6.4	0.233	26.2 ± 7.4	29.4 ± 6.8	0.130
FMI (kg/m²)	11.1 ± 2.1	10.0 ± 2.1	0.022	10.9 ± 2.1	11.3 ± 1.8	0.386
WC *z*-score	2.8 ± 1.2	2.1 ± 1.1	0.012	2.6 ± 1.3	2.8 ± 0.9	0.236
Calcium (mg/dL)	9.9 ± 0.3	9.8 ± 0.2	0.602	9.8 ± 0.2	9.7 ± 0.3	0.746
Phosphorus (mg/dL)	4.5 ± 0.5	4.5 ± 0.7	0.833	4.4 ± 0.4	4.4 ± 0.7	0.832
25(OH)D (ng/mL)	21.8 ± 8.5	24.6 ± 7.4	0.192	22.8 ± 6.3	21.5 ± 6.4	0.138
PTH (pg/mL)	50.9 ± 17.3	48.8 ± 14.9	0.596	52.4 ± 19.3	53.5 ± 15.7	0.798

*Paired *t*-test

*BMI* body mass index, *FMI* fat mass index, *PTH* parathyroid hormone, *WC* waist circumference

**Fig. 2:**
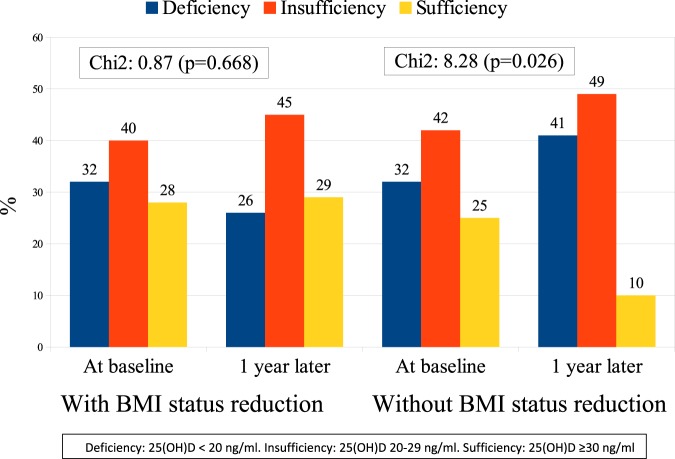
Changes of vitamin D status in obesity group with and without BMI status reduction throughout combined intervention

Figure 2 shows and compares the changes of vitamin D status in the obesity group with and without BMI status reduction throughout combined intervention. There are no changes of vitamin D status in patients with BMI status reduction throughout combined intervention. However, in those patients without BMI status reduction throughout combined intervention, there was a significantly increased prevalence (*P* < 0.05) of hypovitaminosis D along the follow-up period.

## Discussion

Sex, age, season and place of residence have been described as independent factors associated with hypovitaminosis D^1,3,4,7,13,24–26^; however, in this case, no significant differences were detected in the distribution of these factors among the participants included in this study (obese group and control group). This eventuality will make it possible to compare the results obtained without confounding factors.

In this study, the association between childhood obesity and reduced concentrations of 25(OH)D is well established^6,11,27^. Nonetheless, we found that 25(OH)D levels in children with obesity were inversely associated with body fat content, and that this association was stronger than that between 25(OH)D and BMI or body weight^28,29^. On the other hand, patients with obesity exhibited mean PTH values significantly higher than subjects in normal BMI status, a fact that presumably would allow maintaining similar calcium levels in both groups, as we observe in this study. In addition, PTH levels in patients with obesity correlated significantly positively to fat mass, but not significantly to weight or BMI *z*-score.

The low levels of 25(OH)D in patients with obesity could be attributed to decreased active outdoor life and sun exposure^4^. Clinical studies have verified that obesity does not affect the capacity of the skin to produce vitamin D, but could disturb its release from the skin into the circulation. In fact, the increase in blood 25(OH)D concentrations is significantly smaller in the obese than in non obese subjects after sunlight exposure or orally administered vitamin D, possibly due to the decreased bioavailability because of its excessive deposition or “sequestering” in body fat compartments^6^. Liquid chromatography/mass spectroscopy has shown a positive correlation between vitamin D in adipose tissue and serum 25(OH)D. This would indicate that adipose tissue would be a storage site for vitamin D but would not necessarily imply a sequestration^30^. On the other hand, several authors have shown that the levels of bioavailable 25(OH)D (defined as 25(OH)D not bound to vitamin D binding protein) in children with obesity seem similar to children without obesity^27^. According to the free-hormone hypothesis, vitamin D–binding protein may act as a serum carrier and reservoir, prolonging the half-life of plasma 25(OH)D while at the same time regulating its immediate bioavailability to target tissues^31^. Thus, hormonal activity may be reflected by the amounts of bioavailable vitamin, rather than total serum levels.

PTH concentrations were elevated in children with obesity in our study in concordance with most studies^2,4,7^. Hypovitaminosis D implies a lower absorption of dietary calcium. This fact increases PTH secretion (in order to maintain normal levels of serum calcium), and, ultimately, induces osteoclastic activity and, consequently, a loss of bone mineral density^5,32^. Secondary elevation of PTH has been postulated as an independent predictor of obesity. One hypothesis is that this physiologic increase in PTH levels would increase calcium influx into adipocytes, which leads to increased lipogenesis and potentially reduces catecholamine-induced lipolysis as mechanism for increased fat storage^8,9,33^. Nevertheless, some authors have described comparable levels of PTH despite different levels of 25(OH)D in children with and without obesity^27^.

The analysis of the different factors associated to hypovitaminosis D (sex, age group, season of the year and place of residence) suggests that, in children with obesity, age and sex would not be determining variables of body vitamin D content, but confirms a lower tendency to hypovitaminosis D in summer time and when the residence is rural. This finding would support the hypothesis that the higher trend to present hypovitaminosis D deficiency in children with obesity would probably be related to a more sedentary lifestyle and, consequently, a decreased sun exposure.

As we observe in this study, 25(OH)D concentrations were negatively related to fat mass status, whereas PTH levels were positively related to it, and this relationship presaged that a decrease in BMI status or fat mass could normalize these hormonal alterations, as previously described in children and adults^10,11,34^. This study has confirmed a significant increase in the prevalence of hypovitaminosis D in patients with weight or fat mass gain at the end of follow-up with respect to the beginning of the study. However, those individuals who presented with BMI reduction at the end of follow-up showed no difference in the prevalence of hypovitaminosis in comparison with the onset of the study. It is likely that a greater BMI reduction (in fact, at the end of the study they maintain a BMI *z*-score corresponding to obesity) would had resulted in an improvement of the prevalence of hypovitaminosis D.

On another note, we should remark how PTH levels did not change from the onset to the end of the period of follow-up neither in those patients who did not reduce their BMI status nor in those who did. This eventuality suggests that vitamin D biodisponibility is reduced in obese individuals and, consequently, the administration of vitamin D supplements should be considered in these patients in order to avoid the presumably maintained osteoclastic activity induced by PTH, as well as lipogenesis.

In fact, the Endocrine Society recommends the determination of vitamin D in children with obesity and adolescents as individuals at risk of deficiency, and suggests that children with obesity should receive an increased amount of vitamin D (at least two or three times) to satisfy their requirements^20,21^. In addition, hypovitaminosis D could adversely affect the different non-calcemic functions of vitamin D^5^.

## Conclusions

This study shows that obesity increase prevalence of suboptimal vitamin D status, and a BMI status reduction in children with obesity could be required to at least stabilize vitamin D status. In these patients, vitamin D status should be regularly controlled and supplementation may be required.
